# Vertebral fracture as a risk factor for self-harm: a retrospective cohort study

**DOI:** 10.1186/s12891-021-04631-9

**Published:** 2021-09-04

**Authors:** James A. Prior, Fay Crawford-Manning, Rebecca Whittle, Alyshah Abdul-Sultan, Carolyn A. Chew-Graham, Sara Muller, Tom A. Shepherd, Athula Sumathipala, Christian D. Mallen, Zoe Paskins

**Affiliations:** 1 Primary Care Centre Versus Arthritis, School of Medicine, Keele University, ST5 5BG Staffordshire, UK; 2 Midlands Partnership NHS Foundation Trust, Stafford, UK; 3 Haywood Academic Rheumatology Centre, Midland Partnership NHS Foundation Trust, ST6 7AG Stoke-on-Trent, UK

**Keywords:** Vertebral fracture, Self-harm, Primary care

## Abstract

**Background:**

The prevention of self-harm is an international public health priority. It is vital to identify at-risk populations, particularly as self-harm is a risk factor for suicide. This study aims to examine the risk of self-harm in people with vertebral fractures.

**Methods:**

Retrospective cohort study. Patients with vertebral fracture were identified within the Clinical Practice Research Datalink and matched to patients without fracture by sex and age. Incident self-harm was defined by primary care record codes following vertebral fracture. Overall incidence rates (per 10,000 person-years (PY)) were reported. Cox regression analysis determined risk (hazard ratios (HR), 95 % confidence interval (CI)) of self-harm compared to the matched unexposed cohort. Initial crude analysis was subsequently adjusted and stratified by median age and sex.

**Results:**

The number of cases of vertebral fracture was 16,293, with a matched unexposed cohort of the same size. Patients were predominantly female (70.1 %), median age was 76.3 years. Overall incidence of self-harm in the cohort with vertebral fracture was 12.2 (10.1, 14.8) /10,000 PY. There was an initial crude association between vertebral fracture and self-harm, which remained after adjustment (HR 2.4 (95 %CI 1.5, 3.6). Greatest risk of self-harm was found in those with vertebral fractures who were aged below 76.3 years (3.2(1.8, 5.7)) and male (3.9(1.8, 8.5)).

**Conclusions:**

Primary care patients with vertebral fracture are at increased risk of self-harm compared to people without these fractures. Male patients aged below 76 years of age appear to be at greatest risk of self-harm. Clinicians need to be aware of the potential for self-harm in this patient group.

## Background

The prevention of self-harm is an international public health priority [1, 2], but in the UK between 2001 and 2013, there was an observed increase in presentations of self-harm to primary care for both men and women[2]. It is vital to identify populations at risk of self-harm, particularly as this is a risk factor for suicide [3]. The World Health Organisation (WHO) have recommended that non-specialist healthcare professionals could evaluate the self-harm potential of patients who present with symptoms of chronic pain and/or depression [4]. Mental ill-health, notably a history of a depressive disorders or borderline personality disorder (BPD) are risk factors for self-harm [5, 6], and although pain is on the causal pathway for depression, pain is also an independent risk factor for self-harm [7, 8] and is linked with trauma, another prominent risk factor [9]. As such, we posit that patients with osteoporotic fractures (particularly vertebral fractures [10]), have the potential to be more vulnerable to self-harm (defined here as non-suicidal self-harm) due to experiencing many of the same risk factors as patients who self-harm.

Vertebral fractures may result in physical impairments, such as kyphosis and height loss, and have also been linked with psychological sequelae including anxiety [11] and depression [12] As many as 20 % of the elderly population are affected by vertebral fractures. This fracture type has a well-documented association with reduced quality of life and increased disability [13, 14], both of which have been reported as motivators for self-harm in older adult [15]. Furthermore, osteoporosis shares several risk factors with depression, such as smoking, drinking and immobility [16]. Vertebral fractures have also been associated with suicide [17, 18]. Therefore, we hypothesise that the burden presented by such risk factors, either individually or synergistically, in patients with vertebral fracture would increase the risk of subsequent self-harm.

We have recently reported increased risk of self-harm in people with fibromyalgia, rheumatoid arthritis, and osteoarthritis [19], and similarly, the risk of self-harm has previously been examined in osteoporosis and back pain. Using CPRD data, Webb et al., found increased risk of self-harm in females, but not males, with osteoporosis, and in both genders with back pain [7]. They did not however, look at the vertebral fracture population, meaning that risk of self-harm in people with vertebral fractures is still unknown. Our aim was to examine the risk of self-harm in patients with vertebral fractures in a primary care population and to examine the role of age and gender on such risk.

## Methods

### Study design

Using the Clinical Practice Research Datalink (CPRD), we undertook a matched retrospective cohort study using anonymised Read-coded (clinical coding system used in UK primary care) patient data [19]. CPRD is a database of primary care records covering around 7 % of the UK population. This coded consultation and prescription data is representative of the UK population, in relation to sex, age and ethnicity [20].

### Study Population

We identified patients aged ≥ 18 years with vertebral fracture between 1st January 1990 and 31st December 2016. Sample identified was achieved by specific Read codes, based on and refined from an internal code list repository (www.keele.ac.uk/mrr). An index date was assigned to each patient corresponding to the date of their diagnosis.

A matched unexposed cohort was constructed as a comparison, this included primary care patients without a previous coded diagnosis of vertebral fracture. No exclusions were made based on the presence of other chronic diseases, including osteoporosis. Cases were grouped by gender and 10-year age range and then frequency matched to unexposed patients. Each control was assigned a pseudo-index date, generated at random from between their 18th birthday and study end date. All individuals were subsequently examined for a Read code which identified self-harm. The codes used were based on both the Read code list used in previous CPRD research[2] and a review (by clinical members of the research team) of all self-harm Read codes available for selection within primary care electronic health records (Code list available at www.keele.ac.uk/mrr upon request). Our self-harm definition was based on the presence of any relevant non-suicidal self-harm code and did not specify method of self-harm, all suicide related codes were removed. Patients were excluded if their self-harm code occurred prior to their index date. Incidence was based only on the first self-harm code reported post vertebral fracture.

### Statistical Analysis

Descriptive statistics were used to characterise the vertebral fracture cohort, including age, gender, practice-level deprivation (the socio-economic status of households in a defined geographical area where the practice is located), previous diagnosis for anxiety and/or depression, BMI, smoking status (never/ex-smoker or current) and alcohol consumption (1–9 or ≥ 10 drinks per week) and (the latter three defined by the closest value recorded before the index date). The incidence rate of self-harm per 10,000 person-years (PY) was determined for vertebral fracture from 01/01/1990 to 31/12/2016. Patients contributed data after the latest of three events: (1) the study start date, (2) the date they registered at a participating practice, plus 6 months or (3)the ‘up-to-standard’ date (the practice reached internal quality standards.

Cox proportional-hazards regression analysis was used for the time-period of 1990 to 2016. Crude hazard ratios (HR) were reported with 95 % confidence intervals (CIs) to examine the association between the presence of vertebral fracture and subsequent incidence of self-harm compared to the matched unexposed cohort. Adjusted analysis was then undertaken, accounting for age, gender, smoking status, alcohol consumption, BMI, practice-level deprivation and, anxiety and depression. Cases with missing data for BMI, smoking status and alcohol consumption were included within analysis using a missing category approach to avoid making the assumption they were missing at random, which is unlikely to be the case. This approach provides transparency of data and our analytical approach. Imputation was not considered sensible in this case, because data were unlikely to be missing at random [21]. Schoenfeld’s residuals were examined to determine the proportionality of hazards for each model. Where variables showed evidence of non-proportionality, they were included as time varying covariates. Further analyses were conducted, stratifying by median age of the exposed cohort and gender. We defined our dichotomized age subgroups as those patients < 76.3 years or ≥ 76.3 years. Data were analyzed with Stata software (version 15.1, StataCorp, College Station, TX, USA).

This study is based in part on data from the CPRD, obtained under license from the UK Medicines and Healthcare products Regulatory Agency (MHRA). The data is from patients who have provided informed consent and collected by the NHS as part of their care and support. This study was approved by the CPRD Independent Scientific Advisory Committee (reference number 18_018R3) prior to release of requested data and has adhered to their guidelines.

## Results

### Sample characteristics

The number of cases of vertebral fracture was 16,293, with a matched unexposed cohort of the same size. The mean age in the exposed cohort was 74.8 (similar in the unexposed cohorts) and the majority of patients were female (70.1 %) and. The prevalence of anxiety and depression in the exposed cohort was approximately double that seen in the unexposed (Table 1).

**Table. 1:** Characteristic of patients with vertebral fracture and their matched cohorts (1990–2016)

Factor	Exposed (%)(*n* = 16,293)	Unexposed (%)(*n* = 16,293)	*p*-value
Mean age at index (SD)	74.84 (10.7)	74.31 (11.2)	< 0.001
Females	11,549 (70.1)	11,549 (70.1)	1.000
Median years follow-up (IQR)	4.1 (2.3, 7.1)	4.8 (2.5. 8.7)	< 0.001
Deprivation status			
Q1 (Least deprived)	3763 (23.1)	3059 (18.8)	< 0.001
Q2	2911 (17.9))	2786 (17.1)	
Q3	2947 (18.1)	3335 (20.5)	
Q4	3284 (20.1)	3603 (22.1)	
Q5 (Most deprived)	3388 (20.8)	3510 (21.5)	
BMI (kg/m^2^)			
Underweight (< 18.5)	590 (3.6)	136 (0.8)	< 0.001
Healthy weight (< 18.5–24.9)	6036 (37.1)	2136 (13.1)	
Overweight (25.0-29.9)	4955 (30.4)	2213 (13.6)	
Obese (> 30.0)	2467 (15.1)	1267 (7.8)	
Missing	2245 (13.8)	10,541 (64.7)	
Smoking			
Never/Ex smoked	5656 (34.7)	12,908 (79.2)	< 0.001
Current smoker	962 (5.9)	2373 (14.6)	
Missing	9675 (59.4)	1012 (6.2)	
Alcohol consumption			
Never/Ex-drinker	1719 (10.6)	3881 (23.8)	< 0.001
Current 1–9 drinks per week	3387 (20.8)	7879 (48.4)	
Current > = 10 drinks per week	663 (4.1)	2240 (13.7)	
Missing	10,524 (64.6)	2293 (14.1)	
Anxiety	3350 (20.6)	1690 (10.4)	< 0.001
Depression	4505 (27.7)	2330 (14.3)	< 0.001

### Incidence and risk of self-harm in Vertebral fractures

Overall incidence of self-harm in the cohort with vertebral fractures was 12.2 (95 %CI 10.1, 14.8) per 10,000 PY. When stratified by age, the incidence was 13.4 (95 %CI 10.4,17.1) in those < 76.3 years and 10.8 (95 %CI 8.0, 14.7) in those ≥ 76.3 years. When stratified for gender, incidence was 17.7 (95 %CI 12.6, 23.2) and 10.3 (95 %CI 8.1, 13.2) for males and females respectively (Table 2).

**Table. 2:** Risk of self-harm associated with vertebral fracture

	Exposed		Non-exposed		Hazard ratios (95 % CI)	
Vertebral fracture	n	Incidence rate, per10,000 (95 % CI)	n	Incidence rate, per10,000 (95 % CI)	Crude	Adjusted^a^
*Total*	104	12.2 (10.1, 14.8)	51	5.1 (3.9, 6.7)	**2.3 (1.7,3.3)**	**2.4 (1.5, 3.6)**
Age						
< 76.3	63	13.4 (10.4, 17.1)	25	4.3 (2.9, 6.4)	**3.1 (1.9, 4.8)**	**3.2 (1.8, 5.7)**
=>76.3	41	10.8 (8.0, 14.7)	26	6.1 (4.2, 9.0)	**1.7 (1.0, 2.7)**	1.7 (0.9, 3.3)
Gender						
Male	41	17.1 (12.6, 23.2)	12	4.2 (2.4, 7.3)	**4.0 (2.1, 7.7)**	**3.9 (1.8, 8.5)**
Female	63	10.3 (8.1, 13.2)	39	5.4 (4.0, 7.5)	**1.8 (1.2, 2.7)**	**1.9 (1.1, 3.2)**

^a^adjusted for age, BMI, smoking status, alcohol consumption, anxiety, depression and practice-level deprivation. Bold = statistically significant (*p* < = 0.05)

When associations were examined, there was a significant crude association between vertebral fracture (HR 2.3 (95 %CI 1.7, 3.3)) and subsequent self-harm compared to having no recorded vertebral fracture. This association was retained after adjustment (HR 2.4 (95 %CI 1.5, 3.6) (Table 2). After stratifying at the median age, we found there to be a marked difference between the crude risk of self-harm across the < 76.3 and ≥ 76.3 years strata for the vertebral fractures cohort (HR 3.1 (95 %CI 1.9, 4.8) and HR 1.7 (95 %CI 1.0, 2.7) respectively). This difference in risk of self-harm between the age strata became greater after adjustment and a significant association was only retained for those younger than 76 years (< 76.3 years: HR 3.1 (95 %CI 1.9, 4.8), ≥ 76.3 years: (HR 1.7 (95 %CI 0.9,3.3)).

Crude analysis found males with vertebral fractures to be almost four times more likely to self-harm compared to those without a previous vertebral fractures (HR 4.0 (95 %CI 2.1, 7.7)) and females to be almost two times as likely to self-harm (HR 1.8 (95 %CI 1.2, 2.7)). These significant associations were altered little by adjustment, (Males: HR 3.9 (95 %CI 1.8, 8.5); Females HR 1.9 (95 %CI 1.1, 3.2)) and remained statistically significant (Table 2).

## Discussion

Although the absolute incidence of self-harm was low, we found that primary care patients with vertebral fractures are at increased risk of self-harm compared to matched patients without fractures. Age and gender were also found to be effect modifiers, with patients aged below < 76 years and male strata showing an increased risk of self-harm in those with vertebral fractures.

Overall, in this study, male patients with vertebral fractures showed the greatest risk of self-harm with a 4-fold increase compared to an age matched unexposed cohort. This contrasts with the trend for higher rates of self-harm seen in females in the general population, and the previous study using CPRD which examined risk of self-harm in osteoporosis [2, 7]. This may be explained by differences in the experiences and perceptions of males with vertebral fractures, or in clinical risk factors. Males who display low bone density, and increased fracture risk, often have an underlying cause such as medication use (steroids), hormone related conditions such as hypogonadism or lifestyle behaviours, such as smoking [22–24]. The presence of multimorbidity is associated with higher instances of self-harm and mental illness diagnosis, whilst behavioral factors such as smoking are risk factors for depression in and of themselves [25, 26]. Further, conditions such as hypogonadism which can increase fracture risk are linked themselves, with mental ill-health in men [27]. In a study exploring males experience of having osteoporotic vertebral fractures, osteoporosis was perceived as an old women’s disease [28]. A gendered societal view of fractures relating to osteoporosis/low bone mineral density (BMD) can cause a threat to masculinity, leading to avoidance of seeking help or consulting healthcare practitioners [28–30], disbelief and difficulty discussing with others. This relates to research on males with chronic pain who reported that they avoided seeking health care, and expectations of being ‘stronger’ or more ‘able to cope’ were prevalent in those who self-harmed [31]. Furthermore, men can experience delayed diagnosis and treatment when it comes to osteoporosis, due to health care professional knowledge and gender differences in licensing of anti-osteoporotic medications [32] with males finding this “depressing” and “frustrating” [28].

Age contributed to the risk of self-harm, as those under the age of 76 with vertebral fractures were found to be three times more likely to self-harm as those in the matched population. Webb et al., similarly reported significant odds ratios in those < 60 years with osteoporosis, as well as for cancer, coronary heart disease, stroke, and COPD [7]. Though not explaining the identified association between vertebral fracture and self-harm, several examined covariates (i.e. anxiety and depression) were significantly associated with self-harm. However, there remain other factors which may play a role. Vertebral fractures are known to cause pain which is a risk factor in and of itself for self-harm [8], as is use of some pain medications, and vertebral fractures have been linked with other factors which may mediate the risk, including decreases in health-related quality of life, loss of independence, low self-esteem and social problems [33–35].

### Strengths and limitations

This is the first study to examine vertebral fracture as a risk factor for incident self-harm. Our use of a large UK primary care dataset has allowed us to examine the incidence of self-harm as well as examine the role of age and gender. The accuracy of vertebral fracture codes has previously been validated [35] and our analysis also takes account of clinically recorded depression, a key risk factor for self-harm.

Several limitations to our work do need to be considered. First, our methods do not facilitate the identification of people with uncoded vertebral fractures. Vertebral fractures are often asymptomatic or undiagnosed and do not come to clinical attention [36], with one study reporting 80 % of women with vertebral fracture being undiagnosed post radiographic investigation. Although the majority of vertebral fractures are likely to be a result of low trauma in this age group, the degree of trauma is not recorded. We have also not addressed the question of risk of self-harm in people with osteoporosis and/or other types of fragility fracture; osteoporosis is known to be poorly coded in CPRD with < 30 % of those receiving osteoporosis medication having an osteoporotic code [37] and we hypothesized that of all the fragility fractures, vertebral fractures were more likely to be associated with chronic pain and therefore self-harm risk. We were not able to examine mechanisms in this study; for example, pain is likely to be a key contributory factor in the risk of self-harm [32]; however, it could not be determined from consultation record data. Furthermore, it remains unclear to what extent use of medications and treatments influence the risk of self-harm. Our original intention within this analysis had also been to examine year-on-year incidence and breakdown the age strata as well, as the used strata form wide age groups, but numbers were too small for such analysis. We also found large proportions of missing data for BMI, smoking and alcohol consumption within the CPRD dataset, particularly in the unexposed patients. As such data is not “missing at random”, we were unable to impute these missing values. However, we included missing data as a separate category in our models and reported the extent of missing data in descriptive tables to ensure transparency. Finally, though our adjustment for anxiety and depression would have captured the largest proportion of mental health conditions, there remain conditions we were unable to adjusted for. Importantly this includes BPD, which can be underreported in non-specialist settings [38] and therefore the small sample which reported self-harm meant identifying any related codes within this primary care dataset would be unlikely.

## Conclusions

Although the absolute incidence is low, people with vertebral fractures have increased risk of self-harm compared to matched unexposed patients. Both age and gender are strong effect modifiers, with males aged below 76 years being at increased risk. Further work is needed to explore the mechanisms of this association and develop appropriate interventions. In the meantime, healthcare professionals need to be vigilant, explore mood, assess risk, and offer appropriate support, management and referrals and signposting especially to younger people and males with vertebral fractures.

## Data Availability

This study is based in part on data from the Clinical Practice Research Datalink obtained under license from the UK Medicines and Healthcare products Regulatory Agency. The data is provided by patients and collected by the NHS as part of their care and support. The interpretation and conclusions contained in this study are those of the author/s alone. The data that support the findings of this study are available from CPRD, but restrictions apply to the availability of these data, which were used under license for the current study, and so are not publicly available. Data are however available from the authors upon reasonable request and with permission of CPRD. The copyright of the morbidity definitions lists (©2014) used in this publication is owned by Keele University, the development of which was supported by the Primary Care Research Consortium. The authors would like to acknowledge Keele University’s Prognosis and Consultation Epidemiology Research Group who have given us permission to utilise the morbidity definitions (©2014). For access/details relating to the morbidity definitions lists (©2014) please go to www.keele.ac.uk/mrr.

## Abbreviations

CPRD: Clinical Practice Research Datalink
BMI: Body Mass Index
PY: Person Years
HR: Hazard Ratio
CI: Confidence Interval
BMD: Bone Mineral Density
COPD: Chronic Obstructive Pulmonary Disease

## References

[1] Organization WH. World health statistics 2016 - Monitoring health for the SDG’s. Sustainable development goals.. In.; 2016.

[2] Carr MJ, Ashcroft DM, Kontopantelis E, Awenat Y, Cooper J, Chew-Graham C, Kapur N, Webb RT. The epidemiology of self-harm in a UK-wide primary care patient cohort, 2001–2013. BMC Psychiatry. 2016;16:53. 10.1186/s12888-016-0753-5 PMC4770684 26923884

[3] Runeson B, Haglund A, Lichtenstein P, Tidemalm D (2016). Suicide risk after nonfatal self-harm: a national cohort study, 2000–2008. J Clin Psychiatry.

[4] Organization WH. Assessment for self harm/suicide in persons with priority mental, neurological and substance use disorders. In.; 2020.

[5] Fliege H, Lee JR, Grimm A, Klapp BF (2009). Risk factors and correlates of deliberate self-harm behavior: a systematic review. J Psychosom Res.

[6] Levy KN, Johnson BN: Personality disorders. In J. C. Norcross, G. R. VandenBos, D. K. Freedheim, & N. Pole (Eds.), APA handbooks in psychology®. APA handbook of clinical psychology: Psychopathology and health (p. 173–207). American Psychological Association. In.; 2016.

[7] Webb RT, Kontopantelis E, Doran T, Qin P, Creed F, Kapur N (2012). Risk of self-harm in physically ill patients in UK primary care. J Psychosom Res.

[8] Theodoulou M, Harriss L, Hawton K, Bass C (2005). Pain and deliberate self-harm: an important association. J Psychosom Res.

[9] Johnson BN, Lumley MA, Cheavens JS, McKernan LC (2020). Exploring the links among borderline personality disorder symptoms, trauma, and pain in patients with chronic pain disorders. J Psychosom Res.

[10] Society NO. Life with osteoporosis: the untold story. Key findings from research into the realities of life with osteoporosis. In.; 2014.

[11] Gold DT (2001). The nonskeletal consequences of osteoporotic fractures. Psychologic and social outcomes. Rheum Dis Clin North Am.

[12] Cizza G, Primma S, Coyle M, Gourgiotis L, Csako G (2010). Depression and osteoporosis: a research synthesis with meta-analysis. Horm Metab Res.

[13] Group WHOS. The burden of musculoskeletal conditions at thestart of the new millenium. Institutional Repository for Information Sharing:1-218. In.; 2003.

[14] Cauley JA, Thompson DE, Ensrud KC, Scott JC, Black D (2000). Risk of mortality following clinical fractures. Osteoporos Int.

[15] Troya MI, Babatunde O, Polidano K, Bartlam B, McCloskey E, Dikomitis L, Chew-Graham CA (2019). Self-harm in older adults: systematic review. Br J Psychiatry.

[16] Clarke DM, Currie KC (2009). Depression, anxiety and their relationship with chronic diseases: a review of the epidemiology, risk and treatment evidence. Med J Aust.

[17] Chang CF, Lai EC, Yeh MK (2018). Fractures and the increased risk of suicide: a population-based case-control study. Bone Joint J.

[18] Jang SY, Cha Y, Kwak JH, Kim KJ, Kim HY, Choy WS (2020). What Is the Difference in the Risk of Suicide Death Between Spine Fracture in Patients Older Than 65 Years and Matched Controls? A Large-database Study from South Korea. Clin Orthop Relat Res.

[19] Prior JA, Paskins Z, Whittle R, Abdul-Sultan A, Chew-Graham CA, Muller S, Bajpai R, Shepherd TA, Sumathipala A, Mallen CD (2021). Rheumatic Conditions as Risk Factors for Self-Harm: A Retrospective Cohort Study. Arthritis Care Res (Hoboken).

[20] Herrett E, Gallagher AM, Bhaskaran K, Forbes H, Mathur R, van Staa T, Smeeth L (2015). Data Resource Profile: Clinical Practice Research Datalink (CPRD). Int J Epidemiol.

[21] Sterne JA, White IR, Carlin JB, Spratt M, Royston P, Kenward MG, Wood AM, Carpenter JR (2009). Multiple imputation for missing data in epidemiological and clinical research: potential and pitfalls. BMJ.

[22] Briot K, Roux C (2015). Glucocorticoid-induced osteoporosis. RMD Open.

[23] Golds G, Houdek D, Arnason T (2017). Male Hypogonadism and Osteoporosis: The Effects, Clinical Consequences, and Treatment of Testosterone Deficiency in Bone Health. Int J Endocrinol.

[24] Izumotani K, Hagiwara S, Izumotani T, Miki T, Morii H, Nishizawa Y (2003). Risk factors for osteoporosis in men. J Bone Miner Metab.

[25] Morgan C, Webb RT, Carr MJ, Kontopantelis E, Chew-Graham CA, Kapur N, Ashcroft DM (2018). Self-harm in a primary care cohort of older people: incidence, clinical management, and risk of suicide and other causes of death. Lancet Psychiatry.

[26] Toprak S, Cetin I, Guven T, Can G, Demircan C (2011). Self-harm, suicidal ideation and suicide attempts among college students. Psychiatry Res.

[27] Ilias I, Alesci S, Gold PW, Chrousos GP (2006). Depression and osteoporosis in men: association or casual link?. Hormones (Athens).

[28] Minns Lowe CJ, Toye F, Barker KL (2019). Men’s experiences of having osteoporosis vertebral fractures: a qualitative study using interpretative phenomenological analyses. Osteoporos Int.

[29] Solimeo SL (2011). Living with a `women’s disease’: risk appraisal and management among men with osteoporosis. J Mens Health.

[30] Solimeo SL, Weber TJ, Gold DT (2011). Older men’s explanatory model for osteoporosis. Gerontologist.

[31] Taylor B. Exploring the perspectives of men who self-harm. Learning in Health and Social Care 2003:83–91.

[32] Group NOG. NOGG 2017: clinical guideline for the prevention and treatment of osteoporosis. In.; 2017.

[33] van Schoor NM, Smit JH, Twisk JW, Lips P (2005). Impact of vertebral deformities, osteoarthritis, and other chronic diseases on quality of life: a population-based study. Osteoporos Int.

[34] Alexandru D, So W (2012). Evaluation and management of vertebral compression fractures. Perm J.

[35] Van Staa TP, Abenhaim L, Cooper C, Zhang B, Leufkens HG (2000). The use of a large pharmacoepidemiological database to study exposure to oral corticosteroids and risk of fractures: validation of study population and results. Pharmacoepidemiol Drug Saf.

[36] Cooper C, Atkinson EJ, O’Fallon WM, Melton LJ (1992). Incidence of clinically diagnosed vertebral fractures: a population-based study in Rochester, Minnesota, 1985–1989. J Bone Miner Res.

[37] Morley J, Moayyeri A, Ali L, Taylor A, Feudjo-Tepie M, Hamilton L, Bayly J (2020). Persistence and compliance with osteoporosis therapies among postmenopausal women in the UK Clinical Practice Research Datalink. Osteoporos Int.

[38] Zimmerman M, Mattia JI (1999). Differences between clinical and research practices in diagnosing borderline personality disorder. Am J Psychiatry.

